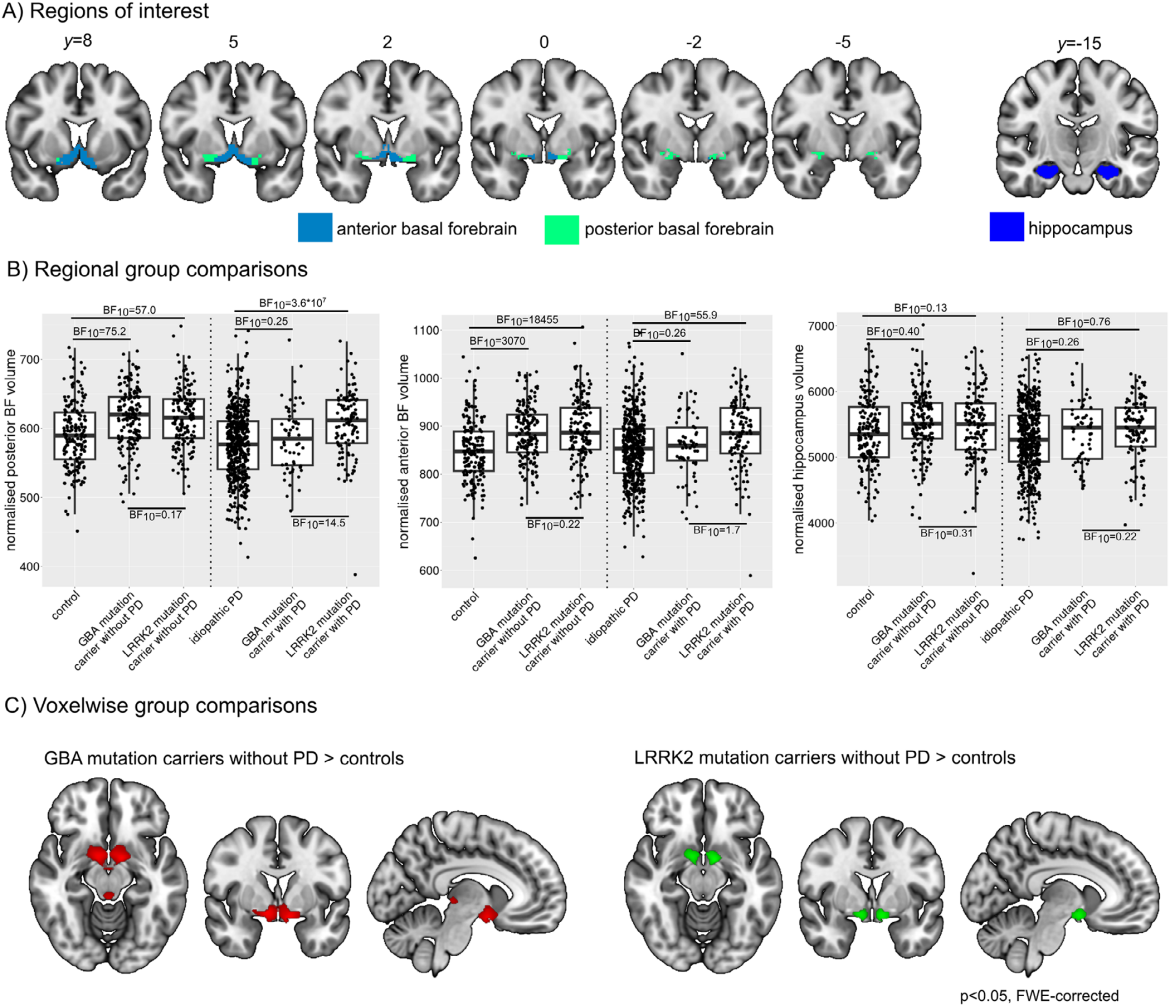# Associations between basal forebrain volume and cognitive decline in GBA and LRRK2 mutation carriers from asymptomatic stages to Parkinson’s disease

**DOI:** 10.1002/alz.091082

**Published:** 2025-01-09

**Authors:** Julia Schumacher, Stefan Teipel, Alexander Storch

**Affiliations:** ^1^ DZNE, Rostock, MV Germany; ^2^ Deutsches Zentrum für Neurodegenerative Erkrankungen e. V. (DZNE) Rostock/Greifswald, Rostock Germany; ^3^ Department of Psychosomatic Medicine, Rostock University Medical Center, Rostock Germany; ^4^ University Medical Center Rostock, Rostock, MV Germany

## Abstract

**Background:**

In people with Parkinson’s disease (PD), mutations in GBA and LRRK2 are associated with different clinical phenotypes which might be related to differential involvement of the cholinergic system. We aimed to investigate cholinergic basal forebrain (cBF) volume in asymptomatic and symptomatic mutation carriers in comparison to idiopathic PD and healthy controls and associations with cognitive decline.

**Method:**

This study included 149 asymptomatic GBA and 169 asymptomatic LRRK2 mutation carriers, 112 LRRK2 carriers and 60 GBA carriers with PD, 492 idiopathic PD, and 180 healthy controls from the Parkinson’s Progression Markers Initiative (PPMI). cBF volumes were extracted using an established automated MRI volumetry approach based on a stereotactic atlas (Figure 1A). We also estimated hippocampal volumes as a control analysis. Bayesian ANCOVAs were used to compare volumes between (1) asymptomatic carriers and controls including covariates for age, sex, and years of education and (2) mutation carriers with PD and idiopathic PD with an additional covariate for disease duration.

**Result:**

We found evidence for an increase in cBF volume in asymptomatic GBA (Bayes Factor against the null hypothesis (BF_10_)=75.2) and LRRK2 mutation carriers (BF_10_=57.0) compared to controls and for no group differences between the two mutation groups (BF_10_=0.17) (Figure 1B). At the PD stage, cBF volumes were increased in LRRK2 compared to GBA mutation carriers (BF_10_=14.5) and idiopathic PD (BF_10_=3.6*10^7^), with no difference between idiopathic PD and PD‐GBA (BF_10_=0.25). Whole‐brain voxelwise comparisons confirmed that increased volume in asymptomatic mutation carriers was mainly found in the region of the cBF (Figure 1C). There were no group differences in hippocampal volume (Figure 1B).

Over five years, idiopathic PD and PD‐GBA declined across all cognitive domains whereas the PD‐LRRK2 group only declined in processing speed. Linear mixed models revealed a significant interaction between cBF volume and time in predicting multiple cognitive domains in idiopathic PD and PD‐GBA, but not in PD‐LRRK2.

**Conclusion:**

While LRRK2 and GBA mutations are both associated with increased cBF volume at the asymptomatic stage, at the PD stage this increase persists only in LRRK2 carriers and might be related to the slower rate of cognitive decline in these patients.